# Two Cases of Worms in the Vagina and in the Ear

**Published:** 1807-10

**Authors:** F. B. Sayre


*Two Cases of Worms in the Vagina and in the Ear. From the Manuscript Notes of the late* Dr. F. B. SAYRE.

I HAD one case of worms in the vagina. The patient was a child about two years old. There was considerable excoriation of the parts, with a discharge from the vagina much resembling gonorrhœa. Some heat in making water, and a most troublesome itching. The complaint had continued several weeks, when I was called upon. From the nicest inspection, and most attentive consideraiton of these symptoms, I could by no means satisfy myself of the cause; however, as the child was otherwise in good health, I judged topical applications were alone necessary; and accordingly prepared a solution of saccharum saturni, and furnished the mother with a syringe, directing a little of the solution to be thrown into the part, and the excoriations to be washed with it four or five times a day. After using this remedy three days, small worms, resembling the maggots which are usually formed in cheese, began to come away, and continued to be discharged two or three a day, for many days. They were all dead when discharged, and that they came from the vagina I could not doubt, as I took several of them therefrom myself, by means of a probe introduced for the purpose. When the worms were all destroyed, the discharge ceased, and the child was very soon after, perfectly well. This case happened in the year 1795 to a daughter of Mr. Andrew Nickell, in Nottingham, in the county of Burlington.

In the winter of 1794, I was called upon by Samuel Jones, a shoemaker in Crosswicks, county of Burlington, New-Jersey, for my advice in a case of deafness. This man had a discharge of fetid matter from the ear, a troublesome itching in the part, and had frequently taken worms out of the ear. Several more of these came away whilst he was under my care. They were about the size and shape of a grain of rye, of a light brownish colour, with several circular rings round the body. This complaint had been of some months standing when I was consulted, and though several remedies were employed in the way of injection, the worms continued now and then to make their appearance: the discharge was undiminished and the deafness remained. He was under my care about two weeks, during which time, the only benefit he received, was a diminution of pain. One ear was only affected.

